# Construction methods and biomedical applications of PVA-based hydrogels

**DOI:** 10.3389/fchem.2024.1376799

**Published:** 2024-02-15

**Authors:** Yi Zhong, Qi Lin, Han Yu, Lei Shao, Xiang Cui, Qian Pang, Yabin Zhu, Ruixia Hou

**Affiliations:** ^1^ Zhejiang Key Laboratory of Pathophysiology, Department of Cell Biology and Regenerative Medicine, Health Science Center, Ningbo University, Ningbo, China; ^2^ Research Institute for Medical and Biological Engineering, Ningbo University, Ningbo, China; ^3^ Department of Otorhinolaryngology, Lihuili Hospital of Ningbo University, Ningbo, China

**Keywords:** PVA, hydrogels, articular cartilage restoration, electronic skin, wound dressing

## Abstract

Polyvinyl alcohol (PVA) hydrogel is favored by researchers due to its good biocompatibility, high mechanical strength, low friction coefficient, and suitable water content. The widely distributed hydroxyl side chains on the PVA molecule allow the hydrogels to be branched with various functional groups. By improving the synthesis method and changing the hydrogel structure, PVA-based hydrogels can obtain excellent cytocompatibility, flexibility, electrical conductivity, viscoelasticity, and antimicrobial properties, representing a good candidate for articular cartilage restoration, electronic skin, wound dressing, and other fields. This review introduces various preparation methods of PVA-based hydrogels and their wide applications in the biomedical field.

## 1 Introduction

Polyvinyl alcohol (PVA)-based hydrogels are attractive polymeric materials with great potential for biomedical applications. PVA, a synthetic macromolecular polymer, has relatively excellent mechanical properties and is biocompatible, inexpensive, and stable (Wang D. et al., 2023). These advantages make it a prevalent hydrogel preparation material in bioengineering; it is increasingly used to interface with living organisms and function in disease treatment, physiological signal monitoring, and others (Liu et al., 2019).

Benefiting from the numerous hydroxyl side chains on PVA, it can be grafted with different functional groups that may endow it with properties such as elasticity (Fang et al., 2020), antimicrobial properties (Li et al., 2022e), electrical conductivity (Su et al., 2020b), self-healing (Pan et al., 2020), environmental sensitivity (Montaser et al., 2019), and 3D printability (Lim et al., 2018; Abouzeid et al., 2020). Pure PVA hydrogels suffer from mismatched mechanical properties when used in biomedical applications. Therefore, much research has controlled and improved the PVA-based hydrogel structure and function by applying suitable cross-linking methods and adding different component materials to modify and change the hydrogel structure, allowing PVA-based hydrogels to be adapted to multiple applications and making them ideal candidates for cartilage scaffold materials (Liu J. et al., 2020; Yang et al., 2020), electronic sensing (Song et al., 2020; Ye et al., 2020), and wound dressings (Zhao H. et al., 2020; Zou et al., 2020).

A common problem of using synthetic hydrogels in biomedical applications is the mechanical strength and toughness insufficiency (Li et al., 2022b). Many scientists have investigated PVA-based hydrogels with simultaneously improved toughness and ductility by designing the hydrogel molecular structure by enhancing the crystallinity (Liu J. et al., 2020; Ye et al., 2020), filling nanoparticles (Jing et al., 2019; Liu et al., 2021), and building double networks (Li et al., 2019; Li L. et al., 2020). Accordingly, PVA-based hydrogels with the necessary properties and functions for each application can be synthesized through the rational selection and combination of toughening components. This paper reviews the different PVA-based hydrogel cross-linking methods and the latest research on their modification for biomedical applications; many superior mechanical properties of hydrogels are synthesized by combining multiple cross-linking methods. We also provide an outlook on further research directions and application prospects (Lu et al., 2020; Sun et al., 2020; Yang et al., 2020; Zhou et al., 2021).

## 2 Cross-linking methods of PVA-based hydrogels

The cross-linking methods of PVA-based hydrogels can significantly impact the biological and mechanical properties. The main reason is the difference in the type and amount of interaction forces between PVA chains in hydrogels synthesized by different cross-linking methods. Cross-linking methods are divided into physical cross-linking when the PVA chains are connected by non-covalent interactions and chemical cross-linking when connected by covalent bonds. Many hydrogels with superior properties use both cross-linking methods in combination (Sun et al., 2020; Pei et al., 2021; Shao et al., 2023). Table 1 summarizes some attractive preparation methods for PVA-based hydrogels.

**TABLE 1:** The preparation of PVA-based hydrogels and their mechanical properties.

*Composition*	*PVA Concentration (wt%)*	*Type of Cross-link*	*Mathods*	*Tensile Strength (MPa)*	*Tensile Modulus (MPa)*	*Strain (%)*	*Toughness* (*MJ/m* ^*3*^)	*References*
AMPS-QAX CNC/PVA	20	physical	F-T cyclic	0.03	0.01	771	—	*Liu et al. (2020b)*
starch/PVA/IL-AlCl_3_	∼15	physical	F-T cyclic	0.53	—	567	—	*Lu et al. (2022b)*
PVA/PEI(LiCl)	∼18.2	physical	F-T	0.60	—	500	—	*Wang et al. (2020a)*
PVA-TA@talc	15	physical	F-T	0.60	—	700	—	*Pan et al. (2019)*
PVA (DMSO/H_2_O)/CNF-AlCl_3_⋅6H_2_O	∼8	physical	F-T cyclic	0.89	0.29	696	3.54	*Li et al. (2022b)*
LNP/PVA (EG/H_2_O)-AlCl_3_	∼12	physical	F-T	1.24	—	589	—	*Wang et al. (2022e)*
HPC/PVA	16	physical	F-T cyclic & Salting-out	1.30	0.59	520	5.85	*Zhou et al. (2019)*
PVA-PANI	12	physical	Unidirectional freezing	1.46	4.27	416	3.28	*Li et al. (2020a)*
PVA/CMC/TA/MXene	∼11.25	physical	F-T cyclic	1.80	—	740	6.24	*Kong et al. (2022)*
TA@CNC-PVA/gelatin/EG/Al^3+^	∼10	physical	F-T	1.95	—	520	4.15	*Yin et al. (2022)*
PVA (DMSO/H_2_O)-CNF	∼8.25	physical	F-T cyclic & Salting-out	2.10	—	400	4.90	*Ye et al. (2020)*
PVA-HA/PAA/PEG	16	physical	F-T & Annealing	3.71	0.98	381	—	*Chen et al. (2018)*
PVA/SS/Na3Cit	10	physical	F-T & Salting-out	4.42	3.14	478	13.73	*Wang et al. (2022a)*
PVA (DMSO/H2O)	—	physical	F-T cyclic & Salting-out	13.50	—	—	127.90	*Duan et al. (2021)*
PVA (Na2SO4)	10	physical	Freeze-soak in salt solutions	15.00	2.50	2,100	15 0	*Wu et al. (2021)*
BC-PVA-PAMPS	40	physical	F-T cyclic	18.00	181.00	—	—	*Yang et al. (2020)*
PVA-agar (AS)	10	physical	F-T cyclic & salting-out	18.00	7.50	545	42.30	*Sun et al. (2020)*
PVA/HCPE	5	physical	HCPE	98.00	—	550	425.00	*Liu et al. (2021)*
PVA-PB/CNF	—	chemical	Borax	0.00	—	1900	—	*Jing et al. (2019)*
Zn/PVA-PB/NFC	∼1.8	chemical	Borax	0.02	—	605	—	*Chen et al. (2019)*
PVA-PB/PANI@CNF	2	chemical	Borax	0.03	0.03	635	—	*Han et al. (2019a)*
PVA-PB(CNT-CNF)	2	chemical	Borax	0.05	—	317	—	*Han et al. (2019b)*
PVA-PB (CNFs–PPy)	2	chemical	Borax	0.06	—	600	262.90	*Ding et al. (2018)*
PVA/PEDOT:PSS	∼6.7	chemical	GA	0.07	—	239	—	*Zhang et al. (2020)*
Multi CNC-PANI/PVA-PB	12	chemical	Borax	0.17	—	1,085	—	*Song et al. (2020)*
Ag/TA@CNC/PVA-PB	10	chemical	Borax	0.25	—	4,106	—	*Lin et al. (2019)*
PVA-TA-GaIn(NaCl)	∼8	physical & chemical	F-T & Borax	1.13	—	—	1.90	*Zhou et al. (2021)*
PVA-PMR-NaCls	10	physical & chemical	Chemical cross-linkers & Salt immersion	5.61	2.16	600	15.92	*Han et al. (2022)*
PVA/GO-TA-CaCl2	∼16	physical & chemical	F-T & Annealing & Borax	14.38	—	450	27.93	*Cao et al. (2022b)*

Abbreviations mentioned on the table: AMPS, 2-acrylamido-2-methylpropane sulfonic acid; QAX, quaternary ammonium xylan; CNC, coated cellulose nanocrystals; IL, ionic liquid (1-ethyl-3-methyl imidazolium acetate); PEI, polyethyleneimine; TA, tannic acid; DMSO, dimethyl sulfoxide; CNF, cellulose nanofiber; HA, hydroxyapatite; LNP, lignin nanoparticle; EG, ethylene glycol; HPC, hydroxypropyl cellulose; PANI, polyaniline; CMC, carboxymethyl-cellulose; PAA, polyacrylic acid; PEG, polyethylene glycol; SS, silk sericin; Na_3_Cit, sodium citrate; BC, bacterial cellulose; PAMPS, propanesulfonic acid sodium poly (vinyl salt); AS, ammonium sulfate; HCPE, small multi-amine molecules; PB, borax; NFC, nanofibrillated cellulose; CNT, carbon nanotube; PPy, polypyrrole; PEDOT:PSS, poly (3,4-ethylenedioxythiophene): polystyrenesulfonate; GaIn, gallium-indium; PMR, 4-(methacryloyloxy) ethyl-1- phenylene acid and 1,4-(5-hexenyloxy) benzene.

### 2.1 Physical cross-linking

Molecular linkages between physically cross-linked PVA-based hydrogels are mainly conducted by physical entanglement, hydrogen bonds, and hydrophobic and electrostatic interactions. Hydrogels synthesized through weak interaction forces are generally soft, flexible, and self-healing, with no toxic cross-linker residues; therefore, they are favored in the biomedical field. The common physical cross-linking methods of PVA-based hydrogels are mainly freeze-thawing (F-T) cyclic, salting-out, and gradient low-temperature.

#### 2.1.1 F-T method

**FIGURE 1:**
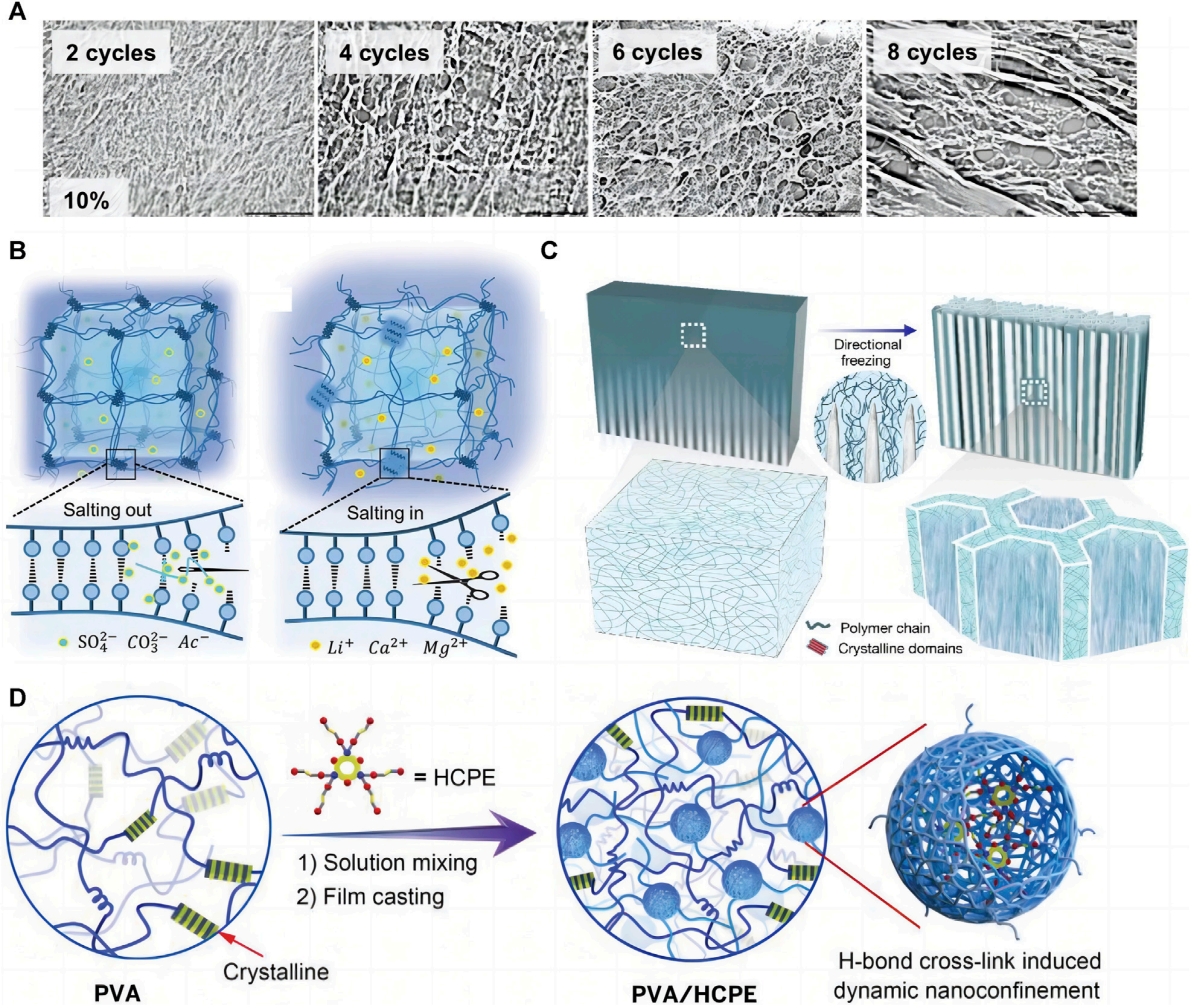
Physical cross-linking methods for PVA-based hydrogels. **(A)** PVA hydrogel prepared by F-T method, the number of F-T cycles can affect the structure and properties of the hydrogel (Stauffer and Peppast, 1992); **(B)** Principle of salting method (Wu et al., 2021); **(C)** Principle of directional freezing method (Hua et al., 2021); **(D)** Cross-linking by hydrogen bonding cross-linker (Liu et al., 2021).

Since Stauffer and Peppast (1992) proposed the PVA-based hydrogel preparation by F-T cyclic in 1991, the method has been widely used in biomaterials, such as wound dressings (Kumar et al., 2020), electronic skin (Zhou et al., 2021), flexible sensors (Yin et al., 2022), and bio-capacitors (Li L. et al., 2020). When hydrogels are prepared with F-T cyclic, PVA chains are discharged due to water molecule crystallization during the freezing process, forming a high-concentration aggregation zone in which the PVA molecular chains are in close contact with each other, forming microcrystalline regions, with physical entanglement and hydrogen bonding between the PVA chains (Holloway et al., 2011). The loose water molecules between the PVA chains are precipitated, increasing the physical entanglement number and hydrogen bonds during thawing and refreezing. This process is repeated, forming a hydrogel filled with water molecules in a three-dimensional mesh structure. The number of F-T cycles, freezing temperature, and time affect the hydrogel molecular structure and mechanical properties (Stauffer and Peppast, 1992), allowing for the control of these properties. The increasing F-T cycle number increases the hydrogel crystallinity, toughness, and tensile properties while decreasing the swelling coefficient (Figure 1A). Our previous studies showed that after about six F-T cycles, the mechanical properties do not change with increasing the F-T cycle number (Hou et al., 2015).

#### 2.1.2 Salting-out method

The salt precipitation method is based on the Hofmeister effect when the hydrogel precursor is immersed in salt solutions such as sodium chloride (Gao et al., 2020), sodium citrate (Duan et al., 2021; Yan G. H. et al., 2022), and ammonium sulfate (Sun et al., 2020), the solvent in the hydrogel precursor is replaced by the salt precipitation effect, aggregating the PVA chains and forming extensive physical cross-linking sites. The high ionic strength of the salt ions reduces the polymer solubility (Baldwin, 1996), further aggregating the PVA chains and causing more hydrogen bonds to form between the PVA chains. The approach enhances the hydrogel mechanical strength and fatigue fracture resistance due to the increased PVA-based hydrogel crystallinity that forms a dense polymer network (Gao et al., 2020). According to Wu et al. (2021), changing the salt type and concentration can adjust PVA hydrogel mechanical properties on a large scale. The higher the anion valence, the better the gelation and toughening effect promotion, while the opposite is true for the cation. The mechanical properties of PVA hydrogels after treatment with different salt solutions show a pattern that follows the Hofmeister effect. He et al. also demonstrated that PVA hydrogels treated with a saturated Na_2_SO_4_ solution showed highly tough and stretchable properties (Figure 1B).

Due to the increase in cross-linking density, the movement and crystallization of water molecules bound in the hydrogel are inhibited, so the hydrogel prepared by this method tends to have better anti-freezing and moisturizing ability. Furthermore, as salt ions are introduced into the PVA network, they can impart ionic conductivity to the hydrogel (Sun et al., 2020; Wang et al., 2021; Han et al., 2022). Many researchers have combined the application of binary solvents to overcome the limited use of ion-conductive hydrogels under low-temperature conditions, preparing cold-resistant electronic skins and sensors with superior performance (Ye et al., 2020; Duan et al., 2021).

#### 2.1.3 Directional freezing

Directional freezing is a common and simple method to prepare anisotropic biomaterials (Hua et al., 2021; Mredha and Jeon, 2022) by applying a gradient low temperature to a hydrogel precursor in a certain way that causes the ice crystals to grow directionally along the temperature gradient; the PVA is discharged into the space between the ice crystals. The lamellar structure of ice crystals is used as a template to form an ordered microcrystalline structure (Zhao et al., 2017) (Figure 1C). Directional freezing facilitates PVA chain alignment and folding into the microcrystalline region, increasing PVA molecular structure ordering and forming a stronger hydrogel network, which can simultaneously improve the hydrogel mechanical strength and properties (Li L. et al., 2020). Most natural biomaterials exhibit anisotropic structures (Yan M. Z. et al., 2022), such as cellulose, lignin, *etc.* While artificial polymers are mostly isotropic. Therefore, increasing research aims to fabricate anisotropic hydrogels by various methods. Anisotropic hydrogels have excellent properties such as strong mechanical strength, cell guidance, tissue regeneration, and anisotropic mechanical/electrical/magnetic/thermal properties (Mredha and Jeon, 2022), allowing their applicability in many fields; stiffness gradient PVA hydrogels prepared with gradient cryogenics can be a tool for basic research on cell adhesion and migration behavior (Kim et al., 2015). The cyclic durability and specific capacitance of ordered polyaniline (PANI)-PVA capacitors prepared by directional freezing are much higher than those of the disordered version (Li W. et al., 2020; Chen Q. et al., 2021). Directional freezing is also important in tissue engineering, as it enables obtaining scaffold materials with unidirectional porous structures of controlled pore size (Yeon et al., 2022). Unidirectional micron-sized pores allow cells to grow inward and spread across the scaffold, while nanopores within the polymer walls of the scaffold allow cell signaling, nutrient delivery, excretion removal, and other vital activities. In addition, the stratified tissue porosity obtained by cryocasting the scaffold facilitates *in vivo* neovascularization and hydroxyapatite-rich cement line formation in the early osteoconduction stages (Zhao N. F. et al., 2020). Zhao et al. (2017) developed a bidirectional freezing scheme by introducing a low thermal conductivity polydimethylsiloxane wedge. This polymer forms a dual horizontal and vertical temperature gradient during cooling, controlling the ice crystal nucleation and growth toward the monolayer direction; the group prepared graphene oxide/PVA films with superb mechanical strength and toughness by this method. Directional freezing is widely used to prepare biomaterials due to the high operability and the obvious optimization of synthetic material properties. In addition, mechanical training was shown to enable hydrogels to acquire anisotropy (Yan M. Z. et al., 2022; Wang Q. et al., 2022).

#### 2.1.4 Hydrogen bonding cross-linker

Dynamic hydrogen bonding substantially connects PVA chains in physically cross-linked hydrogels; hydrogen bonding cross-linkers can increase the hydrogen bonding donors and acceptors in the hydrogel and act as bridging points between the PVA chains. Therefore, the PVA chains are connected and entangled with each other through the hydrogen bonds, resulting in a dense polymer chain network. Typical hydrogen bonding cross-linkers include carbon nanofibers (Li et al., 2022b), tannins (Li et al., 2022c), dopamine, and polyamine molecules (Liu et al., 2021); they are characterized by possessing high strength and abundant hydroxyl binding sites, which can enhance the interactions between the chains through many strong hydrogen bonds and lead to polymer chains with a high surface density. PVA-based hydrogels prepared with potent hydrogen-bonding cross-linkers provide high toughness, ductility, and tensile strength simultaneously (Liu et al., 2021; Li et al., 2022b) (Figure 1D) due to the dynamic fracture and reorganization of hydrogen bonds in the hydrogel network when they are subjected to tensile stress, which effectively dissipates energy and endows these hydrogels with good self-healing properties (Lin et al., 2020). The hydrogels prepared by this cross-linking method are biocompatible, only require simple mixing to form a gel, facilitate customizing and integrating special functions such as anti-freezing and electrical conductivity (Liu X. et al., 2022), mass production, and processing, besides being used in manufacturing electronic skin (Wen et al., 2021; Zhou et al., 2021), flexible sensors (Li et al., 2022c), wound dressings [35], and other tissue engineering materials (Pei et al., 2021).

### 2.2 Chemical cross-linking

Chemical cross-linking connects the molecular chains of PVA hydrogels by covalent or metal-ligand bonds; chemical hydrogels have better stability and toughness than physical hydrogels (Xiang et al., 2020). Cross-linking chemical agents facilitate the hydrogel insolubility in water (Ebhodaghe, 2020) and can be glued quickly *in situ* (Yuan et al., 2019; Chen Y. et al., 2021; Wang R. B. et al., 2022), enhancing hydrogel applications in tissue engineering. This is because *in situ* gel formation allows hydrogels to be used as filler materials and bioinks for fine surgical procedures and 3D printing of bionic scaffolds that need adaption to the wound surface (Abouzeid et al., 2020; Wu et al., 2022). Therefore, chemical cross-linking methods are favored by researchers despite the difficulty of completely removing the residual cross-linking agents (Peppas et al., 2006; Zhang et al., 2020; Chen Y. et al., 2021).

#### 2.2.1 Chemical cross-linkers

**FIGURE 2:**
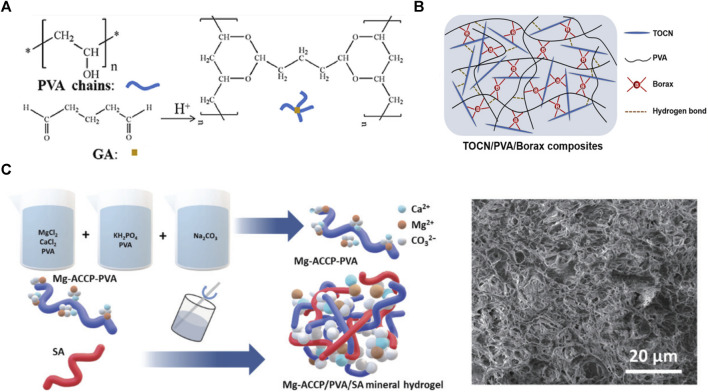
**(A)** PVA-based hydrogel cross-linked by glutaraldehyde (Pei et al., 2021); **(B)** PVA-based hydrogel cross-linked by borax (Songfeng et al., 2020); **(C)** PVA-SA copolymer hydrogel (Shen et al., 2022).

Three main types are the most commonly used chemical cross-linking agents for preparing PVA-based hydrogels. The first is Aldol reaction initiation using aldehydes or ketones with α hydrogen atoms, such as glutaraldehyde, modified starch double aldehyde starch (DS) (Guo et al., 2020; Cao J. L. et al., 2022) (Figure 2A). The hydroxyl group (-OH) of PVA reacts with the aldehyde group (-CHO) of glutaraldehyde in acidic solutions [e.g., sulfuric (Guo et al., 2020) and phytic acids (Wei et al., 2021)] to form acetals or hemiacetals. This method can obtain a hydrogel with good structural stability under relatively mild conditions. Moreover, it possesses mechanical properties such as superior toughness and elasticity (Zhang et al., 2020) and has promising applications in flexible sensing and wound dressing (Hosseini and Nabid, 2020; Wei et al., 2021). However, glutaraldehyde affects cellular activity (Amiri et al., 2022); therefore, the non-toxic, biodegradable, and widely used chemically active modified double aldehyde starch (DS) is proposed as a substitute for glutaraldehyde (Cao J. L. et al., 2022).

The second common cross-linking agent is borates, which polymerize PVA chains through borate ester bonds (Songfeng et al., 2020; Hamd-Ghadareh et al., 2022) (Figure 2B). Borax hydrolysis yields B(OH)_4_^-^, which forms diol-borate bonds with the widely present intermolecular hydroxyl groups of PVA. The reversible covalent bond can be broken and regenerated reversibly when damaged. Such dynamic bonds give PVA/borax hydrogels excellent self-healing properties, super-elongation, and plasticity (Ke et al., 2023). When the hydrogel pH value changes or in a sugar-containing environment (e.g., glucose and fructose), the borate ester bond is reversibly dissociated and reorganized. Monosaccharides such as glucose and fructose are rich in diol units that competitively break the borate ester bond between PVA and borate ions; sugar concentration regulates this destruction degree. Therefore, PVA hydrogels synthesized by this method possess a pH/sugar responsiveness (Yi et al., 2023). Finally, the hydrogel possesses underwater self-healing and conductive properties because the borate ions produced by borate ionization can move in the aqueous medium. These superior properties make PVA/borax hydrogel promising for wearable device applications (Zhang Z. et al., 2022; Li et al., 2022d).

In addition, multivalent metal ions such as Ca^2+^, Mg^2+^, Fe^3+^, *etc.*, Have been used as cross-linkers for PVA-based hydrogels. The metal ions can form coordination bonds with the hydroxyl groups in PVA, constraining the PVA chains, and stabilize the network of PVA-based hydrogels (Shen et al., 2022). The mechanical strength and toughness of PVA-based hydrogels were increased due to the sacrificial breaking of the coordination bonds (Han et al., 2023; Li et al., 2023). In addition, the introduction of metal ions restricts the crystallization behavior of water molecules in the PVA-based hydrogel at low temperatures, thus the hydrogel acquires antifreeze properties (Shen et al., 2022).

#### 2.2.2 Ultraviolet (UV) radiation, electron beam, and gamma irradiation

The cross-linking process by UV radiation, electron beam, or gamma irradiation generates free radicals and induces covalent bond formation between functional groups (Jin, 2022). Cross-linking in these ways requires no chemical cross-linking agent (Nasef et al., 2019), with the advantages of chemically cross-linked hydrogels, i.e., stable structure, superior mechanical properties, and controllable shape. The PVA-based hydrogels prepared by electron irradiation possess high fracture strength, but their cytocompatibility and tissue affinity are relatively lacking; therefore, it is commonly used to manufacture industrial conductive materials (Wang Z. Y. et al., 2023). The PVA-based hydrogels cross-linked by UV and gamma radiation possess significant advantages over the other chemical cross-linking methods mentioned above. Cross-linking is characterized by a mild reaction temperature, pH, and controlled reaction time and space (Eghbalifam et al., 2015; Vo et al., 2022). The properties of hydrogels synthesized by this method have a large scope for adjustment, and various parameters can be used to modify the hydrogel polymerization behavior (e.g., radiation dose, UV exposure, photo-initiator concentration, monomer chain length, and various biomolecule conjugation) (Montaser et al., 2019; Nasef et al., 2019). The PVA-based hydrogels prepared by this cross-linking method possess good mechanical toughness and biocompatibility. The tensile fracture strength, elastic modulus, and toughness reached 1.28 MPa, 426.4 kPa, and 2.53 MJ m^–3^. Hydrogel cross-linking increased the breaking stress, modulus of elasticity, and toughness of the hydrogel by 23, 20, and 25 times, respectively (Pei et al., 2021). PVA-based hydrogels can obtain controlled biodegradation rates by photopolymerization, enabling their wide use in various fields of tissue engineering.

### 2.3 Multi-network copolymerization

Multi-network copolymerization is an attractive strategy for hydrogel synthesis (Ding et al., 2018; Shen et al., 2022; Qin et al., 2023) (Figure 2C). The mechanical and biological features of a single PVA network are constrained by its loose structure, limiting its hydrogel capabilities. Cross-interconnection of multiple networks can enhance the hydrogel structure stability and mechanical properties (Wang et al., 2021; Li et al., 2022a). During macromolecular polymer copolymerization, the hydrogel structure is reinforced by electrostatic interactions, physical entanglement between chains, and extensive hydrogen bonding between functional groups (Wen et al., 2020). Preparing hydrogels by mixing with natural polysaccharides such as sodium alginate (SA) (Shen et al., 2022), chitosan (Shamloo et al., 2021), and starch (Lu L. et al., 2022) can overcome the natural polysaccharide hydrogel brittleness and improve the PVA hydrogel swelling properties and mechanical strength (Qing et al., 2021; Cao J. L. et al., 2022). This allows PVA-based hydrogels to have advantages over natural polysaccharide polymers, such as biocompatibility, biodegradability, and self-healing properties (Ounkaew et al., 2020; Cao J. L. et al., 2022). PVA-based hydrogels achieve excellent electrical conductivity when copolymerized with conductive materials such as acrylic acid (Dai et al., 2022) and PANI (Jing et al., 2019). Therefore, PVA-based hydrogels have potential applications in flexible sensing. Copolymerization with natural polysaccharide derivatives, such as modified double-formaldehyde starch and chitosan, can replace chemical cross-linking agents. In these methods, tough multi-network hydrogels are obtained by esterification or Schiff base reactions; such PVA-based hydrogels are more suitable for biomedical applications than cross-linking by glutaraldehyde (Altaf et al., 2020; Ounkaew et al., 2020; Takacs et al., 2022).

## 3 PVA-based hydrogels for biomedical applications and their construction

### 3.1 Artificial cartilage

**FIGURE 3:**
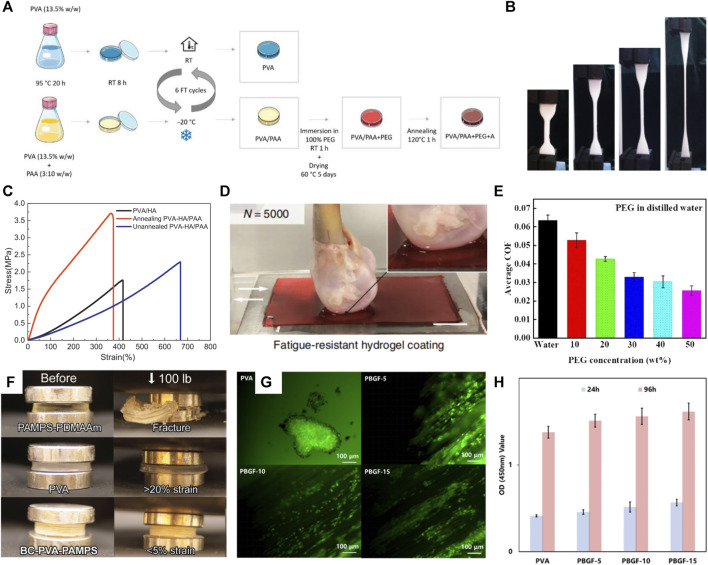
**(A)** Schematic diagram of PVA-based hydrogel synthesis (Branco et al., 2022); **(B)** PVA-based hydrogel has good flexibility; **(C)** Annealing can enhance the mechanical strength of hydrogel (Chen et al., 2018); **(D)** PVA-based hydrogel can be used as articular cartilage contact surface with good tribological properties (Liu J. et al., 2020); **(E)** Organic solvent impregnation can significantly reduce the friction coefficient of hydrogel (Hu et al., 2022); **(F)** PVA-based hydrogel has good compression resistance and has the potential to be used as a load-bearing joint (Yang et al., 2020); **(G**,**H)** PVA-based hydrogel has good biocompatibility (Zhu C. K. et al., 2022).

Articular cartilage is a crucial lubricating and weight-bearing structure in the body. The lack of vascular tissue to transport nutrients makes it difficult for damaged articular cartilage to self-heal. Traditional treatments to promote cartilage self-healing, such as chondrocyte transplantation and microfracture, have low treatment efficiency and long recovery times (Ye et al., 2022; Zhao et al., 2022). Therefore, artificial joint cartilage replacement has become an important treatment modality. An alternative approach is localized articular surface replacement with conventional orthopedic materials (cobalt-chromium alloy, ultrahigh-molecular-weight polyethylene), but these implants have a high degree of stiffness that may ultimately result in abnormal stress and wear on the joints (Yang et al., 2020). The PVA hydrogel is an ideal candidate for preparing bionic articular cartilage (Hu et al., 2022; Oliveira et al., 2023) because of its similar porous structure to that of articular cartilage. Besides the advantages of non-toxicity and good biocompatibility (Figure 3G), it has good permeability and low friction (Figure 3D). The unique physical cross-linking method makes blending with other polymers or functional components possible by simple mixing, as it enables PVA-based hydrogels to have the mechanical strength and necessary biological properties as load-bearing materials.

#### 3.1.1 Mechanical properties enhancement

Articular cartilage has extreme strength (4–20 and 0.8–25 MPa compressive and tensile strengths, respectively (Li et al., 2022a)), toughness (fracture energy of 1,000–15,000 J m^-2^), and elasticity (fracture strain of 60%–120%), and can transmit 7–9 times the body weight, while the very low coefficient of friction (0.001–0.04) makes it possible to load the human body with flexible life activities for a long time (Cao J. L. et al., 2022). In contrast, the ultimate tensile strength of the pure PVA hydrogel was 0.6 MPa, the compressive strength was 24 MPa, and the strain at break was 400% (Ye et al., 2020). And the low compression modulus (0.31–0.8 MPa) of PVA hydrogels causes them to exhibit substantial deformation (>20%). This significant deformation suggests that using PVA alone as synthetic cartilage in the knee joint will cause forces to be transferred to the surrounding bone and cartilage (Yang et al., 2020). Consequently, insufficient mechanical properties are the main obstacle to applying pure PVA hydrogels in bionic joints. Therefore, many studies have developed PVA-based hydrogels with mechanical strength and low friction coefficient (Cao J. L. et al., 2022; Ye et al., 2022). Ye et al. (2022) introduced polyethylene glycol (PEG) into PVA hydrogel and prepared PVA/PEG composite hydrogel by blending the physical cross-linking method; the mechanical properties of the hydrogel were 21.2 MPa, and the friction coefficient was only 0.12.

The PVA-based hydrogels applied to bionic cartilage are mainly formed by the physical cross-linking method through F-T cycles. Consequently, the hydrogel mechanical properties can be effectively enhanced by increasing the hydrogel crystallinity and constructing multi-network hydrogels.

##### 3.1.1.1 Increasing crystallinity

Increasing the number of F-T cycles and annealing treatments can significantly enhance the PVA-based hydrogel mechanical strength (Cao J. L. et al., 2022). Chen et al. (2018) showed that annealing treatment can significantly increase PVA-based hydrogel crystallinity, reduce water content, and form a more dense and stable three-dimensional mesh structure. This treatment substantially increased the tensile strength and elastic modulus of the PVA hydrogel (Oliveira et al., 2023). Annealed PVA-based hydrogels have a low friction coefficient advantage due to dual-phase lubrication and fluid loading support (Chen et al., 2018; Branco et al., 2022). Solvent impregnation was used to enhance the crystallinity of PVA-based hydrogels. This method can effectively improve the mechanical and tribological properties of hydrogels; impregnation with acetone (Hu et al., 2022) and PEG (Branco et al., 2022) reinforced the structure of PVA-based hydrogels, bringing the hydrogels closer to the flexibility of natural cartilage.

##### 3.1.1.2 Building a secondary network

Introducing a flexible second network enhances hydrogel mechanical strength primarily through electrostatic interactions and many hydrogen bond formations that increase the hydrogel cross-link density (Nie et al., 2020). Meanwhile, the sacrificial fracture of the second network can effectively dissipate energy and enhance hydrogel toughness (Yang et al., 2020; Branco et al., 2022) (Figure 3A). Biomaterials such as chitosan (Luo et al., 2022) and bacterial fibers (Yang et al., 2020; Zhu C. K. et al., 2022; Oliveira et al., 2023) are widely used in the second network of PVA-based hydrogels, as they have enhanced the hydrogel structure (Figure 3F). Ye et al. (Cao J. L. et al., 2022) introduced graphene oxide-tannic acid (TA) sub-network structure into PVA-based hydrogels. The tensile strength and fracture toughness of the constructed composite hydrogels reached 11–26 times that of pure PVA hydrogels (14.38 MPa/27.93 MJ m^-3^).

Additionally, a study combined hydrogel with titanium alloy to prepare a “soft (hydrogel)-hard (Ti6Al4V)" integrated material, revealing a very high interfacial toughness (3900 J m^-2^) with a high load-bearing capacity and excellent tribological properties (Ye et al., 2022).

#### 3.1.2 Improvement of other performance

The effects of wear and tear are one of the main problems posed by artificial joints. To ensure the service life of artificial joint cartilage, a wear-resistant hydrogel with a low friction coefficient has become a current research direction. The main methods currently used to improve the tribological properties of hydrogels are lubricants, organic solvent impregnation, and hydrogel surface modification. Hu et al. (2022) encapsulated carbon quantum dots (CDs) in PVA hydrogels and significantly reduced the PVA-based hydrogel friction coefficient. Organic solvent dehydration treatment can also improve the hydrogel abrasion resistance. Ye et al. (2022) showed that the PVA/PEG composite gel could be made to have lubrication and high load-bearing capacity by acetone impregnation to maintain a low COF of 0.12 even under dry friction, and the wear rate was reduced to the original 27.4%. Chen et al. (2018) showed that annealing treatment could reduce the PVA-based hydrogel friction coefficient by about 40% (Figure 3C). Constructing microtextures by laser ablation improved the PVA-based hydrogel surface tribological properties (Zhou et al., 2022).

Introducing bioactive components (such as magnetic composite nanoparticles hydroxyapatite (Hou et al., 2015), hydroxyapatite (Abouzeid et al., 2020), and polysaccharides (Hou et al., 2013)) can promote cell adhesion and proliferation and improve the biological properties of PVA-based hydrogels (Nie et al., 2021). In previous studies, our group has designed nano-hydroxyapatite/PVA composite hydrogels, revealing that hydroxyapatite enhanced the nanocomposite hydrogel compressive strength and promoted cell adhesion and proliferation (Hou et al., 2015). Biodegradable glass (BGF) enhances the mechanical properties of PVA-based hydrogels and promotes cell proliferation and differentiation due to spontaneous BGF degradation, promoting cartilage repair (Zhu C. K. et al., 2022). Additionally, hybrid hydroxyapatite with high osteoconductive properties coated on the surface of PVA hydrogel promotes *in situ* osteogenesis (Luo et al., 2022).

### 3.2 Electronic skin

Wearable medical and health devices have received much attention recently. Polymer hydrogels with sensing properties are widely used in electronic skin and human-computer interaction; they work in health monitoring and disease diagnosis and treatment processes (Qin et al., 2022). PVA-based hydrogels are viable strain and temperature sensors (Chen S. Y. et al., 2022; Liu H. D. et al., 2022) (Figure 5A).

#### 3.2.1 Sensing characteristics

The conductive hydrogel changes its resistance when it is deformed by pressure or when the temperature or environment changes, which is the basis of its function as a sensor. The main sensors designed based on PVA-based hydrogels are pressure, chemical (Qin et al., 2022) (Figure 5D), and temperature (Liu H. D. et al., 2022). Pure PVA hydrogels showed a low conductivity of 4.24 × 10^−5^ S cm^–1^ (Fan et al., 2020). To enhance the electrical conductivity of hydrogels, researchers have introduced conductive components such as carbon nanoparticles (graphene (Pan et al., 2020), carbon nanotubes (Song et al., 2020) and MXenes (Zhang et al., 2019; He et al., 2020)), nanometallic particles, charged ions (Zhou et al., 2019), and conductive active polymers (PANI (Li L. et al., 2020), and polypyrrole (Abodurexiti and Maimaitiyiming, 2022)) into the hydrogel matrix. Enhanced with improved conductivity sensitivity and stability, PVA-based hydrogels have emerged as exceptional candidates for sensor materials. However, their versatility surpasses expectations. By incorporating directional freezing and incorporating natural anisotropic elements, conductive hydrogels gain the ability to respond deliberately to the direction of stimuli, thus expanding the range of applications for these extraordinary materials.

##### 3.2.1.1 Electrical conductivity

**FIGURE 4:**
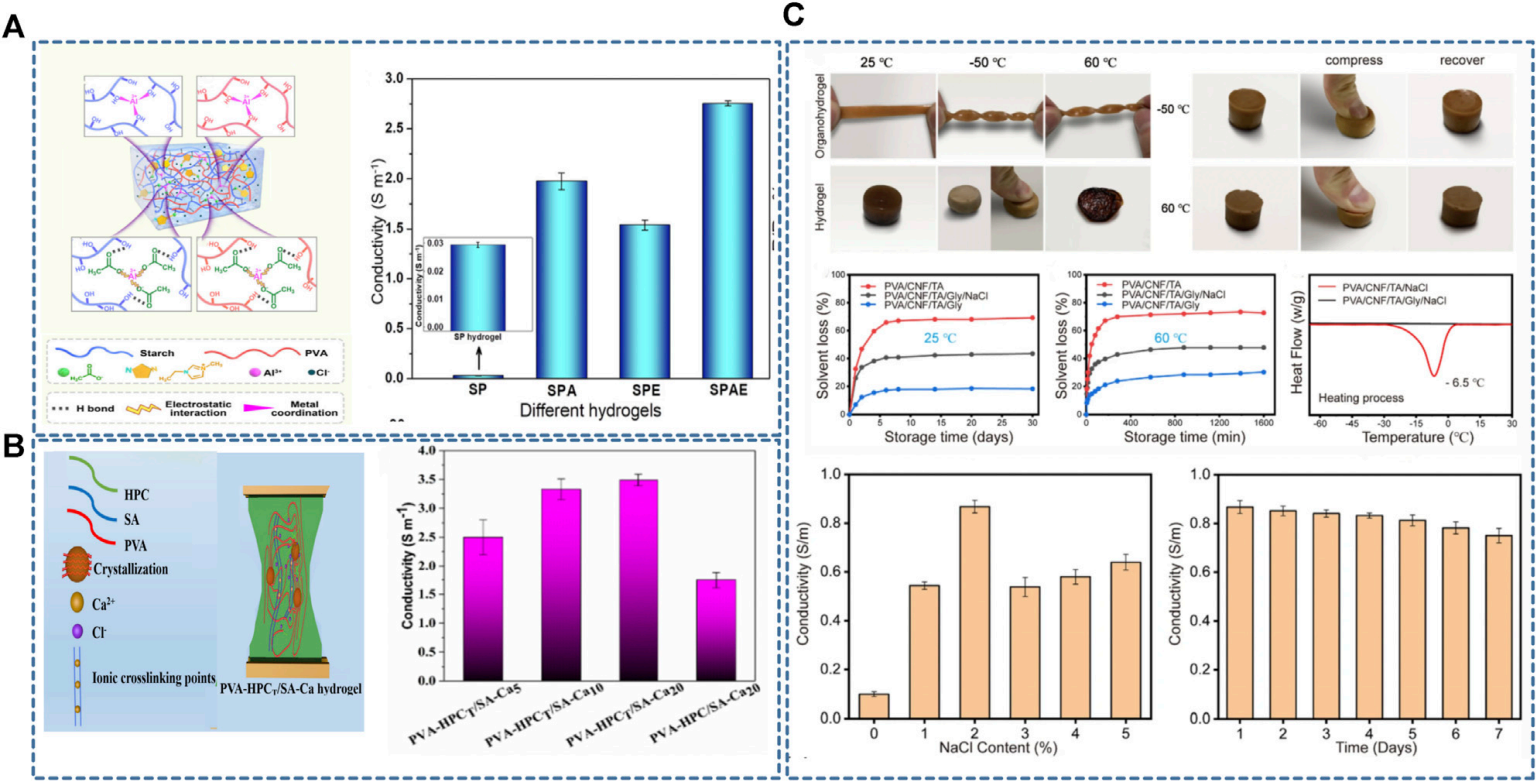
**(A,B)** PVA-based hydrogel doped with Al^3+^ or Ca^2+^, the metal ions can make the hydrogel have good ionic conductivity (Gan et al., 2021; Lu L. et al., 2022); **(C)** The application of organic solvents enables the hydrogel to maintain good electrical conductivity in low-temperature environments and underwater. The concentration of NaCl solution affects the conductivity, and this hydrogel possesses sustained conductivity (Li et al., 2022c).

The current strategies for synthesizing conductive hydrogels include two main directions: Ionic conductive hydrogels prepared by adding electrolytes and electronic conductive hydrogels prepared by filling them with nanoparticles. PVA-based hydrogels possess a three-dimensional porous structure that provides a natural transport channel for the rapid migration of ions in the material. Therefore, introducing metal ions [e.g., Al^3+^(Fan et al., 2020; Lu L. et al., 2022), Ca^2+^ (Gan et al., 2021), Mg^2+^ (Guo et al., 2022), and Fe^3+^ (Merhebi et al., 2020)] in PVA-based hydrogels can form ion-conductive hydrogels (Figure 4A). The electrical conductivity of hydrogels can be improved by enhancing the crystal arrangement orderliness in the hydrogel and improving the migration efficiency of ions in the hydrogel. Ionic conductive hydrogels possess good electrical conductivity, water retention, and frost resistance (Wen et al., 2021). However, the conductive sensitivity (gauge factor value, GF) is relatively low (Lu et al., 2020; Pan et al., 2020). Introducing some metal ions, such as Al^3+^ and Mg^2+^, can enhance the electrical conductivity of hydrogels and make them possess antibacterial properties. The main principles are electrostatic interactions between ions and negatively charged surfaces of bacterial populations and Ph reduction due to Al^3+^ hydrolysis (Lu L. et al., 2022). Introducing various mineral ions can enhance the hydrogel conductivity; Shen et al. (2022) introduced colloidal and amorphous polyionic biominerals (Mg-ACCP, containing Mg^2+^, Ca^2+^, CO_3_^2-^ and PO_4_^3-^) into a biocompatible PVA and sodium alginate network to construct novel hydrogels with abundant mineral ions. This hydrogel has high ionic conductivity and is highly sensitive even to small applied pressures and strains, and its conductivity reaches 1.7 S m^-1^ at a frequency of 10^5^ Hz.

Brine immersion is one of the main methods to impart ionic conductive properties to hydrogels (Han et al., 2022). The all-wood conductive hydrogel obtained by Yan M. Z. et al. (2022) by soaking in ammonium sulfate solution has excellent flexibility, electrical conductivity, and sensitivity. It can accurately distinguish macroscopic or subtle human movements, including finger flexion, pulse, and swallowing behavior, facilitating accurate human motion monitoring. Currently, ionic liquids are receiving great attention due to their high chemical and thermal stability, electrical conductivity, and solubility. Ionic liquids are used to prepare PVA-based hydrogels with high electrical conductivity and frost resistance (Lu L. et al., 2022). Furthermore, copolymerization with conductive polymers, including polyaniline (Qin et al., 2022) and polypyrrole (Abodurexiti and Maimaitiyiming, 2022), can yield excellent conductivity and sensitivity. For example, in 2020, Li et al. achieved an impressive conductivity of 20.5 S/m in a Polyvinyl Alcohol/Polyaniline hydrogel (Li L. et al., 2020).

Zhan et al. (2022) further designed a bilayer Janus conductive hydrogel with conductive and negative or weakly conductive layers, overcoming the mismatch between the mechanical properties of elastomer, the conductive hydrogel, and the lack of strong interfacial adhesion, eliminating the need for insulating elastomers, and making hydrogels more suitable for biomedical applications.

##### 3.2.1.2 Anisotropy

**FIGURE 5:**
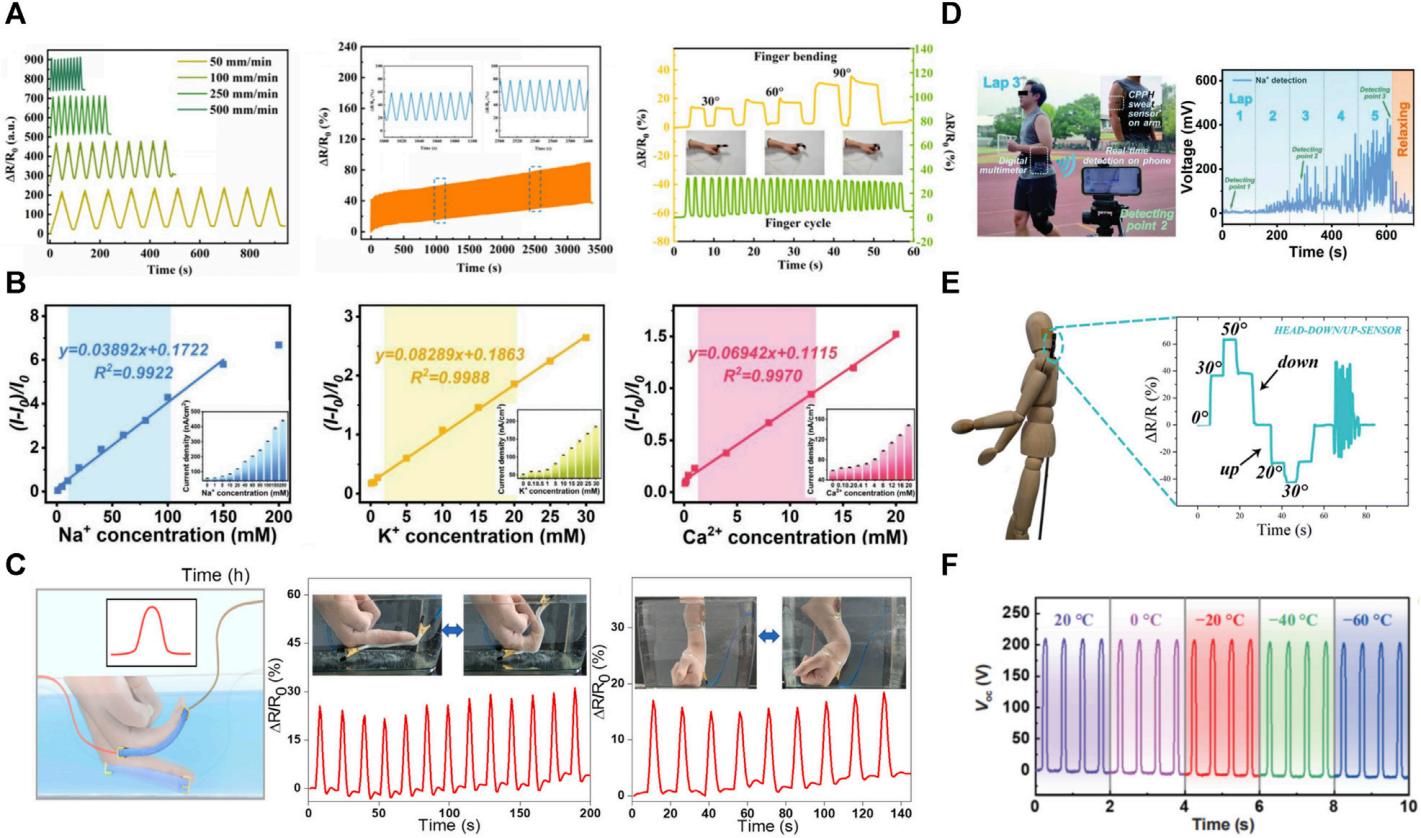
Properties and applications of PVA-based hydrogels in flexible sensing. **(A)** Ionically conductive PVA-based hydrogels can sensitively and reproducibly convert motion signals into electrical signals (Zhang L. et al., 2022); **(B)** Sweat sensors prepared using PVA-based hydrogels can respond to Na^+^, K^+^, Ca^2+^ and their concentrations in sweat (Qin et al., 2022); **(C)**. Anti-freezing and water-preserving design of PVA-based hydrogel can in realizing sensing underwater (Lu L. et al., 2022); **(D)** PVA-based hydrogel monitors the movement process of human body (Qin et al., 2022); **(E)** Anisotropic PVA-based hydrogel can differentiate the movement signals in different directions (Peng et al., 2020); **(F)** PVA-composite hydrogel possesses excellent anti-freezing properties and can realize stable electrical signal conduction at −60°C (Dai et al., 2022).

Direction recognition is another important aspect of electronic skin that realistically mimics human skin, allowing a more accurate determination of the stimulus source. Anisotropic hydrogels are constructed to enable finer and more bionic reading of biosignals by electronic skin; directional freezing is one of the most prominent methods for forming anisotropic biomaterials. Another effective approach is introducing naturally anisotropic components such as cellulose nanofibrils (Yan M. Z. et al., 2022) and lignin (Yan G. H. et al., 2022). The currently proposed direction recognition still has limitations: it can only recognize motions in the same plane, including forward and reverse directions, and more complex motions are difficult to distinguish by electrical signals (Peng et al., 2020) (Figure 5E). Therefore, advanced materials with 3D orientation recognition capability still need to be further explored. It is possible to design the structure of anisotropic hydrogels to obtain three-dimensional woven receptors and then encode the readers to achieve three-dimensional perception.

#### 3.2.2 Mechanical properties

PVA-based hydrogels offer significant versatility in terms of their mechanical properties, enabling adjustment over a wide range. By modifying factors such as the concentration of PVA, the composition of the hydrogel, and the synthesis methods, the mechanical performance of PVA-based hydrogels can be extensively tailored. Therefore, PVA-based hydrogels are well-suited for applications in electronic skin, as they exhibit flexibility, stretchability, and anti-fatigue characteristics. This adjustability allows these hydrogels to meet the diverse requirements of electronic skin (Wu et al., 2021). Progress has been made in toughening hydrogels by forming double networks (Zhang et al., 2023), adding nanofilms, and mechanical training. The main idea is to introduce an energy dissipation mechanism to enhance the fatigue fracture resistance of hydrogels using dynamic bonding, ligand bonding, or sacrificial fracture of the second network (Zheng et al., 2021).

##### 3.2.2.1 Improving the mechanical strength of hydrogels through improved cross-linking methods

The first method to improving the mechanical strength of hydrogels uses a binary solvent. Water is used as a dispersant in conventional PVA-based hydrogels to create high water percentage hydrogels, which leads to loose cross-linking of hydrogels; therefore, reducing the water content enhances the mechanical strength of hydrogel. However, the inherent low solubility of PVA does not support preparing low-water-content hydrogels. Therefore, mixing organic solvents such as ethylene glycol (Wen et al., 2021; Dai et al., 2022), glycerol (He et al., 2020; Ma et al., 2020), and dimethyl sulfoxide with water in a certain ratio can overcome this issue by reducing the water content. Moreover, the strong hydrogen bonds formed between organic solvents and water molecules can enhance the hydrogel flexibility.

The second method Is to add a F-T cycle or a thermal annealing step. As mentioned above, increasing the number of F-T cycles increased physical entanglements and hydrogen bonds in PVA-based hydrogels, producing a stronger PVA-based hydrogel. Thermal annealing could also enhance the mechanical strength of hydrogels prepared by F-T cycles, significantly improving mechanical properties, including fracture toughness and tensile strength (Cao J. L. et al., 2022); hydrogels are subjected to 50°C–120°C and constant humidity for several hours to increase their crystallinity (Chen J. D. et al., 2022).

**FIGURE 6:**
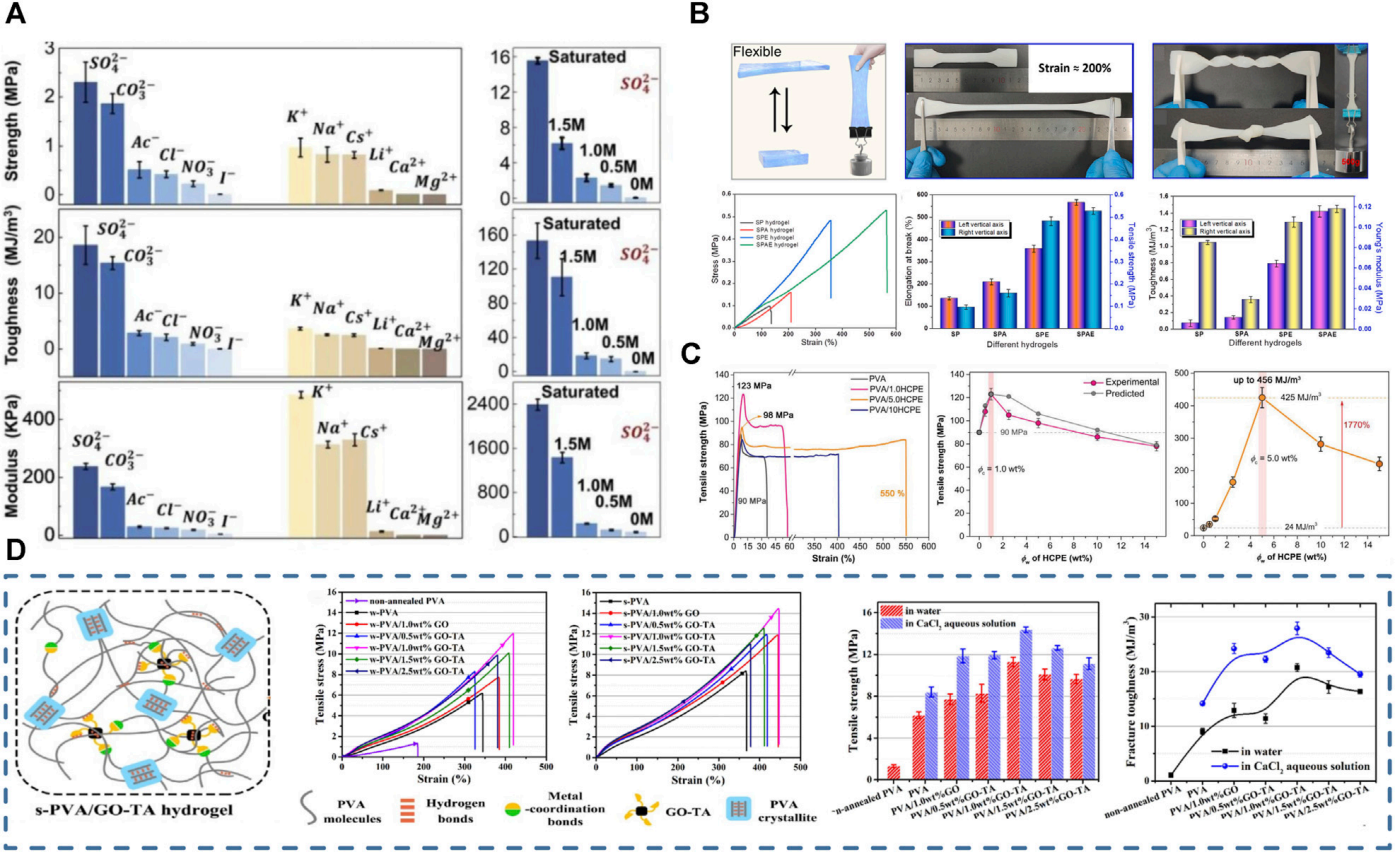
Methods to enhance the mechanical properties of conductive PVA-based hydrogels; **(A)** Salt precipitation, especially immersion in Na_2_SO_4_ solution can substantially enhance the toughness of hydrogels (Wu et al., 2021); **(B)** Starch/PVA dual-network hydrogels possess good tensile properties and tensile strength (Lu L. et al., 2022); **(C)** Small multi-amine molecules enable the formation of dynamic nanoconfinement in the hydrogel, allowing the hydrogel to have excellent mechanical strength (Liu et al., 2021); **(D)** Composite PVA-based hydrogels formed with the introduction of a large number of metal-ligand and reversible bonds possess good mechanical strength (Cao J. L. et al., 2022).

The third is the salting method that effectively enhances hydrogel mechanical strength and toughness (Wu et al., 2021; Yan G. H. et al., 2022) (Figure 6A). Higher concentrations of salt solutions would further enhance the effect on the mechanical properties of hydrogels. Accordingly, the mechanical properties of PVA hydrogels can be adjusted on a large scale by changing the salt type and concentration. The interaction between ions, water molecules, and polymer chains induces different degrees of aggregation of polymer chains, resulting in significantly different mechanical properties. The saturated Na_2_SO_4_ solution-treated PVA hydrogels exhibit considerable strength (15 ± 1 MPa), toughness (150 ± 20 MJ m^-3^), and elongation (2,100% ± 300%) (Wu et al., 2021).

##### 3.2.2.2 Improving the mechanical strength of hydrogels by enhancing PVA networks

The most common approach to improve the hydrogel mechanical strength is to build multiple networks (Figure 6B). The porous structure of PVA-based hydrogels allows them to act as a flexible backbone, and the interpenetrating network increases hydrogen bonding and electrostatic interactions in the material. The multi-network design can effectively improve the mechanical properties of hydrogels (Lu L. et al., 2022). For example, the double network of PVA and sodium alginate has excellent mechanical properties due to chelation between mineral ions and organic matrices (Shen et al., 2022). Additionally, the multi-network synergistic PVA/sodium carboxymethylcellulose/TA/MXene hydrogels show good mechanical strength (1.8 MPa/6.24 MJ m^–3^) (Yan G. H. et al., 2022) (Figure 6A).

Increasing the coordination or reversible bond type and number in the hydrogel network can also enhance its structural stability (Figure 6D). As mentioned previously, carbon nanofibers (Li et al., 2022b), tannins (Mredha and Jeon, 2022; Zhan et al., 2022), dopamine and polyamine molecules (Liu et al., 2021) are widely used as hydrogen bonding cross-linkers for PVA-based hydrogels. F-T-crosslinked hydrogels are more biocompatible than chemically crosslinked ones, although their structure is less stable. Hydrogen bonding is a physical interaction force that allows for tight junctions between PVA molecules–due to the abundant hydrogen bonds–and does not impair the bio-friendly properties of PVA materials. Therefore, the hydrogen bonding cross-linkers enhance the mechanical properties of physically cross-linked PVA-based hydrogels. Using hydrogen bonding cross-linkers can significantly enhance the strength and toughness of hydrogels. For example, Liu et al. (2021) prepared a PVA/HCPE (small multi-amine molecules) composite hydrogel using polyamine molecules, showing excellent fracture strength and strain to 98 MPa and 550% (Figure 6C).

#### 3.2.3 Self-healing and self-adhesive

##### 3.2.3.1 Self-healing

**FIGURE 7:**
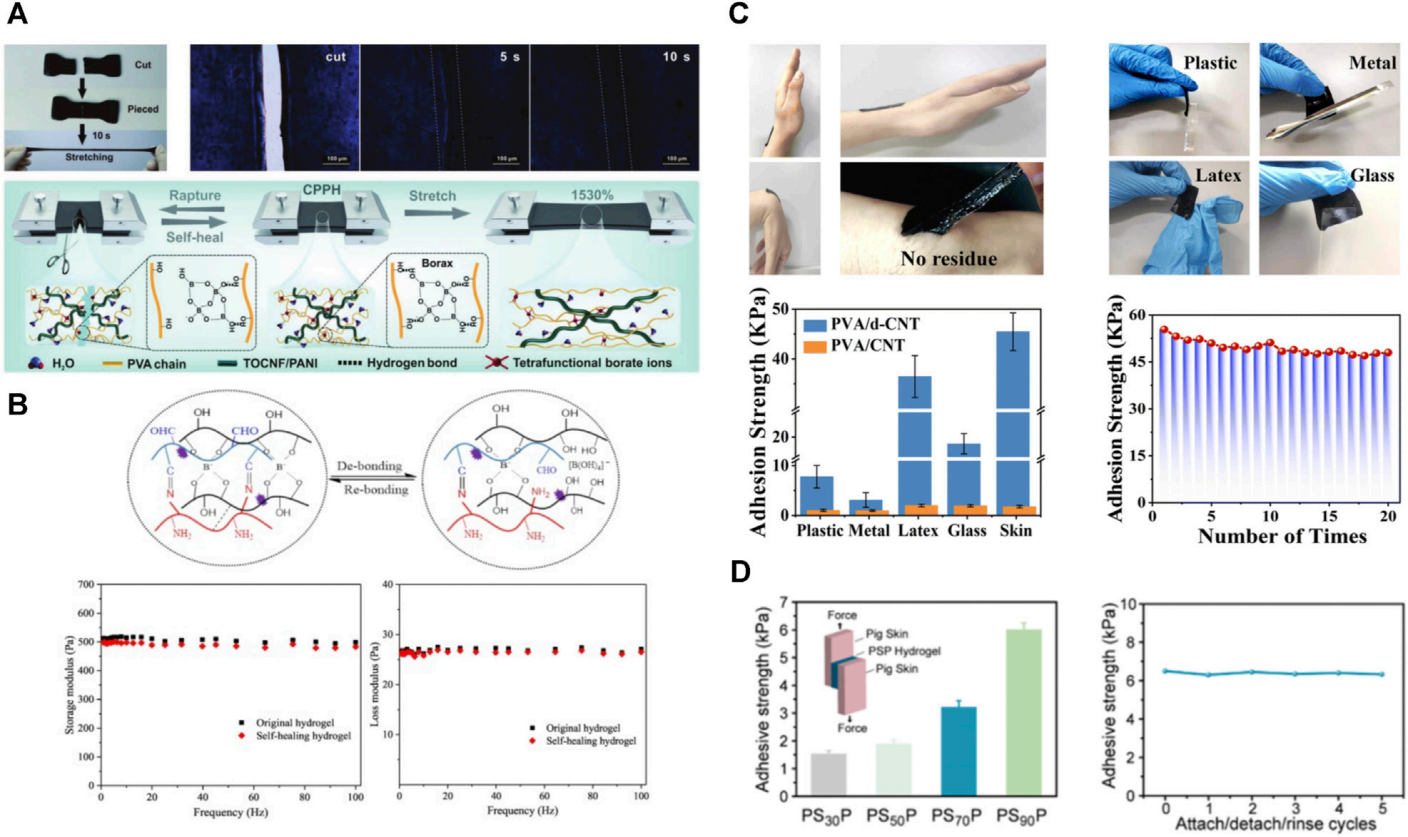
Reversible chemical bonding and non-covalent interactions can confer good self-healing properties to PVA-based hydrogels. **(A)** Self-healing photographs and pattern diagrams of PVA-based hydrogels enriched with borate ester bonding and hydrogen bonding (Qin et al., 2022); **(B)** Schematic illustration of the principle of self-healing in PVA-based hydrogels, and their energy storage modulus and loss modulus before and after self-healing (Cao J. L. et al., 2022); **(C)** Copolymerization with poly (dopamine) enabled the PVA-based hydrogel to obtain excellent adhesion properties, and showed a certain adhesion strength to different substrates (Zhu H. et al., 2022); **(D)** Repeatable interfacial adhesion of the PVA-based hydrogel to the pig skin exhibit (Huang et al., 2023).

Self-healing materials have attracted a great deal of interest from researchers. The ideal self-healing material should be able to restore the mechanical properties and electrochemical functions relatively quickly. PVA-based hydrogels achieve self-healing mainly relying on the reversible breakage and reconstruction of dynamic covalent bonds and supramolecular interactions (Peng et al., 2020). So far, reversible chemical bonds (including disulfide bonds (Li et al., 2022e; Ling et al., 2022), borate ester bonds (Qin et al., 2022), Diels–Alder reactions, and reversible free radical reactions) and non-covalent interactions (including hydrophobic interactions, host-guest interactions, hydrogen bonds, and various weak molecular interactions) have been used to make self-healing hydrogels (Cao J. L. et al., 2022) (Figure 7A). When the material is stressed, dynamic bonds break and reorganize reversibly. Consequently, the material can have beneficial properties such as self-healing, shape memory, and better adaptability, leading to several applications of hydrogels in biomedicine, such as pressure sensors, wound dressings, and tissue adhesives (Takacs et al., 2022). Many polymers connected by non-covalent interactions can also achieve self-healing. However, high temperature or F-T are often required, which prolong self-healing time. Therefore, they can be used for material recycling but do not meet the need for immediate self-healing as electronic skin.

##### 3.2.3.2 Self-adhesive

The tissue adhesion ability of hydrogel is attributed to the chemical/physical bond formed between the hydrogel and the tissue. The amide bond is a usual chemical bond between bonding materials and tissues. The attachment between the aldehyde group of the hydrogel and the amino group of the surrounding tissue occurs because of the abundant presence of amino groups on the tissue surface (Gao et al., 2019). The main way to obtain tissue adhesion in PVA-based hydrogels is by introducing catechol groups or copolymerization with acrylic acid with abundant carboxyl groups (Su et al., 2020a; Zhu H. et al., 2022; Huang et al., 2023) (Figure 7C).

Some technical obstacles limit the development of underwater tissue adhesion hydrogels. First, current underwater adhesives rely mainly on surface patterning (Rao et al., 2018), dense interfacial physical bonding (Wang Z. et al., 2020), surface water absorption with nanocrystal formation or self-hydrophobicity to produce greater physical adhesion to various solid surfaces (Han et al., 2020). Most current underwater adhesives have a higher affinity for non-biological materials such as glass and metal. The second is the difficulty of rapid tissue adhesion and self-healing underwater. Unlike inorganic hydrophilic materials (e.g., glass and metals), animal tissues often contain oils or other hydrophobic substances, greatly reducing the adhesive capacity of most bonded hydrogels (Shao et al., 2018). Hydrophilic groups on the surface of hydrogels and tissues tend to form hydrogen, electrostatic bonds, or both with water molecules and organic components (e.g., glycine) underwater. This can further weaken the hydrogel-tissue and hydrogel-hydrogel interactions (Zhong et al., 2014). The third obstacle is the large swelling rate of most viscous hydrogels in water, which causes significant swelling and embrittlement of the hydrogel network in an aqueous environment. When swelling occurs, the adhesion usually decreases sharply due to the significant decreases in the bonding functional group density.

He et al. reported PVA/tannic acid/carbon nanotubes hydrogel had the ability to adhere pigskin underwater. Due to the presence of a large number of catechol and o-phenyltriol groups in tannic acid, it can endow hydrogel with self-adhesive properties through the formation of hydrogen bonds, electrostatic interactions, and hydrophobic interactions (He et al., 2022). The starch/PVA-borax/tea polyphenol/ethylene glycol hydrogel reported by Tao et al. also showed underwater adhesion properties. The adhesion of this hydrogel is mainly accomplished through the polyphenol chemistry of tea polyphenol, i.e., hydrogen bonding, hydrophobic interactions, metal coordination, and physical interactions (Ke et al., 2023). Other non PVA-based hydrogels, such as acrylamide composite hydrogels, also use hydrophobic properties to obtain underwater adhesion (Han et al., 2020).

#### 3.2.4 Other characteristics

##### 3.2.4.1 Frost resistance and durability

Conventional conductive hydrogels consist mainly of water and are susceptible to environmental factors due to the crystallization of water molecules below zero degrees, which results in a precipitous drop in the hydrogel conductivity at low temperatures. Conversely, hydrogels tend to dehydrate and become dry and hard when exposed to high temperatures. During the working process, the hydrogel gradually loses its original mechanical properties, flexibility, and ionic conductivity due to water loss. Applying binary solvents is one of the main methods to achieve freeze resistance and durability of PVA-based hydrogels. The organic solvents ethylene glycol, glycerol, and dimethyl sulfoxide were introduced into the gel to replace some water molecules (Li et al., 2022c) (Figure 4C). The formation of strong hydrogen bonding interactions between organic solvent and water molecules effectively inhibits forming ice crystals at low temperatures and dehydration at high temperatures. For example, GPPD (Graphene oxide–- PVA/polyacrylamide–- double network) hydrogel was prepared using a binary solvent system of ethylene glycol/water. The device possesses excellent cold resistance and moisture retention properties; it can work stably at very low temperatures of–50°C to–80°C and has a moisture retention duration of 100 days (Dai et al., 2022) (Figure 5F). Strong hydrogen bonding improves the gel stability for use in harsh environments and greatly slows the drying. Chelation between metal ions and organic matrices can also give hydrogels excellent antifreeze properties (Zhu X. Q. et al., 2022; Shen et al., 2022). For example, Mg^2+^ and Ca^2+^ composite hydrogels prepared by Shen et al. demonstrate excellent freeze resistance performance (Shen et al., 2022).

##### 3.2.4.2 Self-powered capability

The self-powered capability allows hydrogel sensors to be adapted to multiple uses, acting as an intrinsic power source and a functional component (Han et al., 2022). Some interesting designs address the energy issues when applying PVA-based hydrogels as flexible sensors. The main designs for flexible wearable devices to achieve self-power are solar cells, friction nanogenerators (Xu et al., 2017; Xu et al., 2023), thermoelectric generators (Khan et al., 2016), and moisture generators (Hu et al., 2021). Among them, friction nanopower (Luo et al., 2021) and water vapor power (Hu et al., 2021) have been practiced in PVA-based hydrogels. Frictional nanogenerators use the friction between the hydrogel and the skin when the joint moves to convert the bending behavior into a voltage signal. Moisture generators are instead implemented by a polymer with ionization groups that spontaneously absorb water molecules from moist air and release positively/negatively charged ions (Luo et al., 2021). For example, Qin et al. (2022) designed a self-powered sweat sensor that enables real-time health monitoring by detecting soluble biomarkers such as electrolytes or metabolites.

### 3.3 Wound dressing

The ideal wound dressing must have passive and active protection to promote wound healing. Passive protection means it fits flexibly over the affected area, insulates the wound from contact with airborne dust and bacteria, and prevents secondary damage from external physical friction. The stretchability of human natural skin is 60%–75%, and the elastic modulus is 5–1,000 kPa in smaller strains (Chen, 2017; Qu et al., 2018), but it can even reach 4.1–18.8 MPa in larger strains (Joodaki and Panzer, 2018). Thus most of the PVA-based hydrogels are suitable for skin applications in terms of tensile properties and elastic modulus. Active protection means equipped with antibacterial and anti-inflammatory features that can slow-release drugs to kill bacteria or promote wound healing. The PVA-based hydrogel structure is similar to that of a natural extracellular matrix, with high water content, flexibility, loose porosity, and other characteristics, representing an ideal candidate for wound dressing materials. The high water content allows the hydrogel to provide a moist environment to the wound and prevent dehydration (Luo et al., 2021). The porous structure and biodegradability of hydrogels facilitate drug delivery, oxygen transport, and slow drug release (Khorasani et al., 2018; Montaser et al., 2019). Accordingly, PVA-based hydrogels are commonly used as wound dressings. The main research directions are to promote the antibacterial and anti-inflammatory effects and achieve conditionally responsive drug release.

#### 3.3.1 Antibacterial and anti-inflammatory

Bacterial infection and inflammation play a crucial role in the development and persistence of chronic wounds. Consequently, it is essential to consider these factors when utilizing hydrogels as wet dressings. PVA-based hydrogels exhibit antimicrobial activity primarily through copolymerization with natural antimicrobial agents, incorporation of antimicrobial components, and application of near-infrared light (NIR) irradiation. The synergistic effect of these various antimicrobial mechanisms has demonstrated notable efficacy.

**FIGURE 8:**
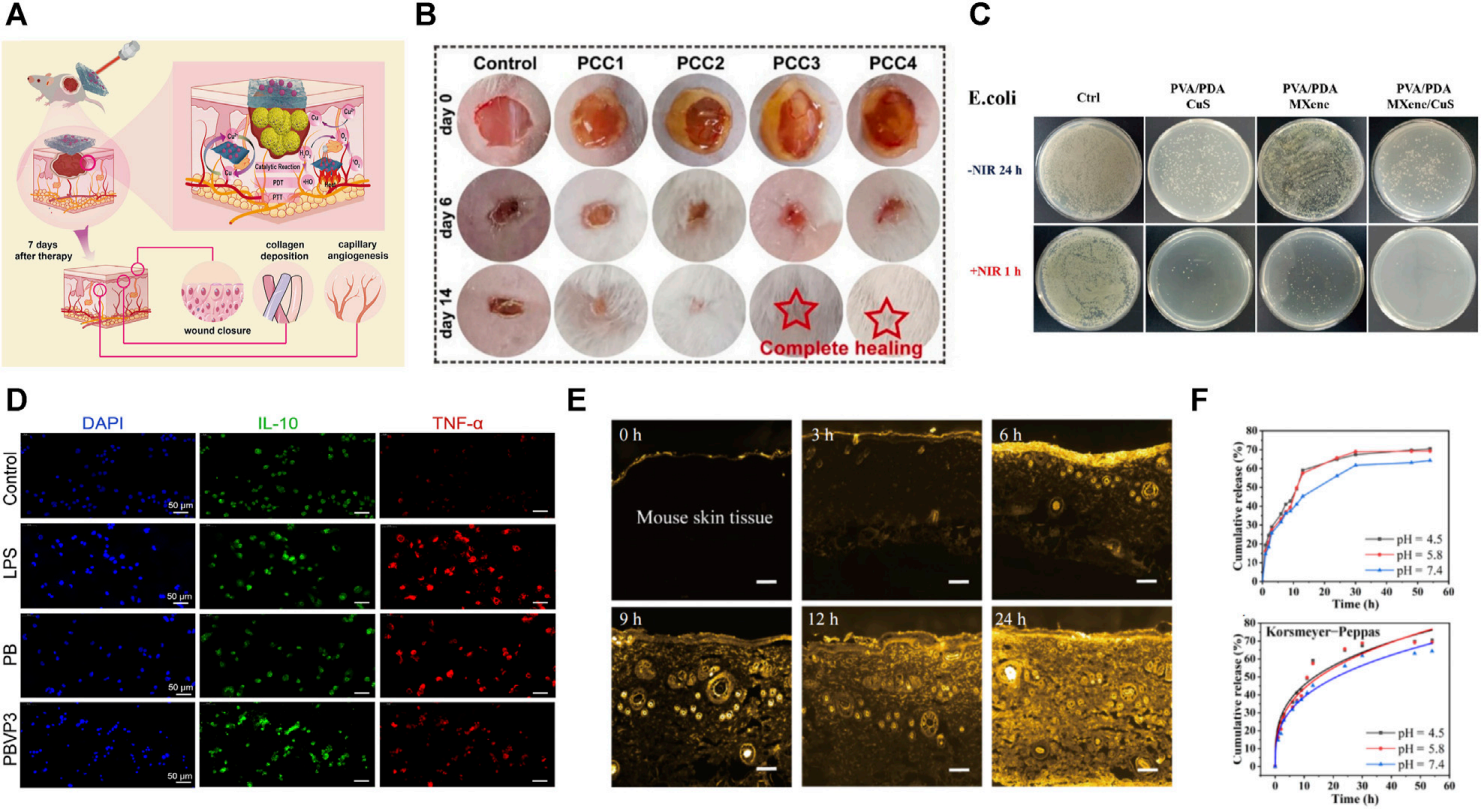
**(A)** PVA-based hydrogels can be affixed to chronic wounds and slowly release drugs under NIR light stimulation (Su et al., 2023); **(B)** PVA-based hydrogels can promote hydrogel wound healing (Liu et al., 2023); **(C)** PVA-based hydrogels can be copolymerized with antimicrobial active materials to obtain better antimicrobial properties (Su et al., 2023); **(D)** PVA-based hydrogels show better anti-inflammatory effects (Liu B. et al., 2022); **(E,F)** PVA-based hydrogels can achieve long-lasting drug release (Chen S. Y. et al., 2022).

The first method is copolymerization with natural antibacterial ingredients. Natural polymers such as chitosan (Shamloo et al., 2021; Cao J. L. et al., 2022; Liu et al., 2023), silk protein (Eivazzadeh-Keihan et al., 2020), and sodium alginate (Abbasi et al., 2020) possess antibacterial activity. Its copolymerization with PVA can give the wound dressing a better healing promotion function and improve its weak mechanical properties (Figure 8B). For example, chitosan-PVA hydrogels have been widely used as trauma dressings, and this composite material possesses excellent antimicrobial activity, and promotes wound healing (Liu et al., 2018). The NH_3_^+^ in chitosan can interact with negatively charged cell membranes, leading to cytoplasmic extravasation. Low molecular weight chitosan can penetrate bacteria and destroy DNA. High molecular weight chitosan can cover the cell surface and inactivate bacteria by depriving them of nutrient supply (Khorasani et al., 2019).

The second method is to load inorganic antimicrobial ingredients. Many inorganic antimicrobial materials have been successfully incorporated into PVA-based hydrogels to synthesize various antimicrobial hydrogels (Wang et al., 2020). It mainly includes metal nanoparticles, metal ions, and carbon nanomaterials. These materials had three main antibacterial mechanisms: the first is that nanomaterials in photocatalysis can produce singlet oxygen (_1_O_2_) and hydroxyl radicals (·OH) to achieve sterilization through catalytic oxidation–photodynamic therapy (PDT) (Wang et al., 2019; Su et al., 2023); nanomaterial Ti_3_C_2_Tx (MXene) (Su et al., 2023), gold nanorods (Qin et al., 2023), and graphene oxide (Wang J. H. et al., 2022) have been widely used for preparing photocatalytic antibacterial dressings (Figure 8C). The second is the use of metal ions to disrupt cell membranes, resulting in the leakage of intracellular substances. Some of these smaller-sized metal elements can pass through the cell membrane and act on the cytoplasm, damaging the bacterial DNA and proteins. Many metallic materials [metallic nanoparticles such as silver (Song et al., 2021; Mojally et al., 2022; Qi et al., 2022; Ke et al., 2023), gold (Qin et al., 2023), cerium oxide (Kalantari et al., 2020), and zinc oxide (Khorasani et al., 2018; Raafat et al., 2018), as well as metal ions such as Cu^2+^ (Xia et al., 2022; Xia et al., 2022), Fe^3+^ (Qi et al., 2022), Al^3+^(Li et al., 2022b; Wang Y. et al., 2022), and Mg^2+^ (Eivazzadeh-Keihan et al., 2020; Albalwi et al., 2022)] have been successfully loaded into PVA-based hydrogels with good antibacterial effects. The third uses the photothermal effect the photothermal agent produces under NIR excitation–photothermal therapy; the principle is that during NIR irradiation, the photothermal agent generates heat, causing a local high temperature and irreversible damage to microorganism protein and enzymes, thus achieving sterilization (Su et al., 2023) (Figure 8A). Many materials with photothermal effects are used for photothermal therapy, such as carbide polymer dots (CPD) (Lu H. J. et al., 2022), graphene oxide (Wang J. H. et al., 2022), and molybdenum disulfide (MoS_2_) (Li et al., 2022e). Qin et al. (2023) designed a PAA/PVA-gold nanorod hydrogel that can rapidly and efficiently disrupt bacterial biofilms and promote infected wound healing under NIR irradiation.

Another method is to load organic antibacterial ingredients. For example, antibiotics (Qiao et al., 2020) [e.g., mupirocin (Zhao H. et al., 2020) and amikacin (Abbasi et al., 2020)], tannins (Li et al., 2022e; Su et al., 2023; Yi et al., 2023), antimicrobial peptides (Zou et al., 2020), and allicin (Wang J. H. et al., 2022) showed good broad-spectrum antibacterial activity in the PVA-based hydrogel bacteria. The design of loaded antimicrobial ingredients suffers from antimicrobial activity loss due to the active antimicrobial ingredient release. Non-releasing antimicrobial materials such as antimicrobial peptides, graphene oxide, chitosan, and ionic liquids can impart sustained antimicrobial activity to hydrogels. Liu B. et al. (2022) designed and synthesized a polymeric ionic liquid-PVA-borax tri-crosslinked hydrogel that achieved long-lasting antibacterial effects due to the non-releasing nature of ionic liquid (Figure 8D).

Furthermore, some studies have promoted wound healing by imparting hydrogel reactive oxygen scavenging (Zhao H. et al., 2020; Qiao et al., 2020) and oxygenation capacity (Li et al., 2022e; Ling et al., 2022; Nour et al., 2022) to slow the inflammatory response of wounds. The MoS_2_@TA/Fe enzyme-anchored multifunctional hydrogel has both advantages: MoS_2_ and TA can scavenge excess free radicals from the affected area, while catalase-like enzymes can decompose H_2_O_2_ to provide O_2_ to the affected area (Li et al., 2022e).

#### 3.3.2 Drug delivery and conditional responsive release

The high water content, good biocompatibility, and the ability to easily encapsulate hydrophilic drugs make PVA-based hydrogels a desirable drug release system (Yao et al., 2022) (Figure 8E). The drug loading and delivery rate are mainly related to the hydrogel microstructure and swelling. Molecular imprinting is used to prepare hydrogels, which can be tailored to the hydrogel microstructure by controlling their polymerization behavior, facilitating hydrogel preparation for efficient and targeted drug release (Kakkar and Narula, 2022). The specific microstructure allows the hydrogel to respond to changes in the wound microenvironment or to the production of a certain substance. Hydrogels that exhibit responsiveness to pH, light, temperature, and other biological signals have garnered considerable attention (Qin et al., 2023).

PVA-based hydrogels can achieve environmentally responsive drug release through reactive cross-linking agents. For example, a ROS-scavenging hydrogel using ROS-responsive linker cross-linking was designed by Zhao H. et al. (2020), which downregulated pro-inflammatory cytokines, upregulated M2 phenotype macrophages, and promoted angiogenesis and collagen production. Meanwhile, due to the responsive cutting of the cross-linker, the hydrogel gradually degrades and releases loaded mupirocin and granulocyte-macrophage colony-stimulating factors, which inhibit bacterial infection and accelerate wound repair, respectively.

Part of the design achieves responsive release of the drug by copolymerizing PVA with a bio-signal responsive material. For example, sodium alginate, rich in carboxyl groups, can accept or release loaded anti-inflammatory drugs depending on the environmental pH. PVA/SA hydrogels have successfully controlled pH-sensitive drug release (Montaser et al., 2019; Abbasi et al., 2020). Qiao et al. (2020) developed a smart hydrogel-based trauma dressing by physically cross-linking PVA with an UV-cleavable polymer. The hydrogel can monitor bacterial infections by detecting specific substances they release and treating infections by stimulating antibiotic release through NIR. Qin et al. (2023) prepared a photothermal nanocomposite hydrogel that responds to NIR stimulation by exploiting the photothermal effect of gold nano-ions. The hydrogel could rapidly eliminate over 80% of Gram-negative/positive bacteria under NIR irradiation.

### 3.4 Other applications

PVA-based hydrogels are widely used in various biomedicine fields due to their good biocompatibility and suitable and tunable mechanical properties, showing great potential for applications in long-acting drug delivery, treatment, and chronic disease diagnosis.

**FIGURE 9:**
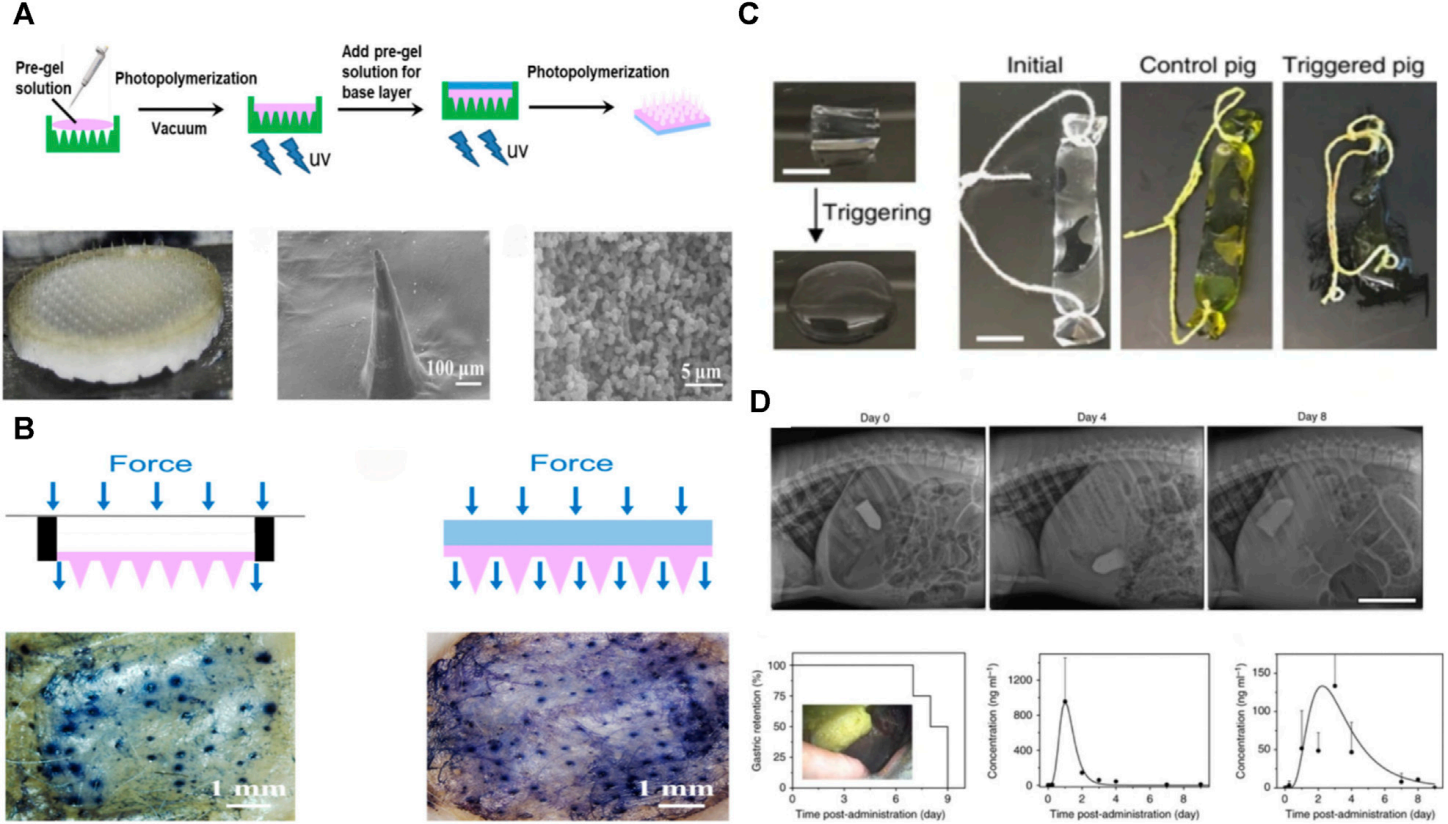
Other applications of PVA-based hydrogels; **(A)** Schematic and photographs of the synthesis of PVA-based hydrogels for microneedle patches; **(B)** Schematic and photographs of microneedle patch application (Chen S. Y. et al., 2022); **(C)** PVA-based hydrogel for gastric retention device, which can be rapidly dissolved in the stomach and retained for a long period of time up to 30 days (Liu et al., 2019); **(D)** Gastric retention device in porcine stomach with X-ray photographs and drug release profile (Liu et al., 2017).

Hydrogels have been designed as skin microneedle patches due to their water-absorbing properties and ease of transition between relatively rigid and flexible states (Wu et al., 2021). Microneedle (MN) based transdermal drug delivery systems are gaining interest as an alternative to traditional vaccine, drug, and cosmetic delivery methods. Chen S. Y. et al. (2022) designed a borate-PVA hydrogel for efficient skin penetration and sustained glucose-responsive insulin delivery (Figure 9A).

Patients often need to take medications continuously for an extended period during chronic disease treatment; a carrier for a long-acting, sustained-release drug can simplify the treatment process, facilitate chronic disease management, and help improve patient compliance with medication. For example, Sunita et al. loaded PVA-based hydrogels with calcipotriol to treat psoriasis by adding polyvinylpyrrolidone to PVA to improve its hydrophilicity and water permeability (Thakur et al., 2023). The results show that the hydrogel can achieve prolonged drug release, promote epithelial tissue regeneration, and heal psoriatic lesions.

Hydrogels are ideal for gastric retention materials, enabling *in vivo* physiological monitoring, diagnostics, and extended drug delivery (Bellinger et al., 2016; Liu et al., 2017). A gastric retention device was prepared by Liu et al. (2019) that utilized polyacrylic acid particles with high water absorption to encapsulate a PVA-based hydrogel. It can rapidly absorb water and swell and reside in the stomach of the animal for a long time. The porous and loose structure of PVA-based hydrogels makes loading drugs easier, while adding nanocrystalline domains makes them robust, resilient, and fatigue-resistant. This is the core principle of the ability to perform therapeutically and remain robustly in the stomach under repetitive mechanical compression and gastric acid erosion (Figure 9C).

A hybrid coupling consisting of a PVA-coated Aβ probe has high sensitivity, selectivity, biocompatibility, and economy; this nanoprobe can enable the early diagnosis of Alzheimer’s disease by detecting Aβ peptides in human serum (Hamd-Ghadareh et al., 2022). In addition, PVA-based hydrogels have also been applied in areas such as crystalline lenses (Ran et al., 2023) and vascular pathways (Mannarino et al., 2020).

## 4 Summary and prospect

Due to the unique structure of PVA, PVA-based hydrogels have multiple promising applications in biomedicine. The researchers adopted different methods to make PVA-based hydrogels obtain various properties. These methods have their limitations and cannot adapt to all situations. For example, due to the low temperature and annealing, the physical cross-linking method for preparing hydrogel cannot be used for cell-loading research. Preparing the hydrogel by chemical cross-linking introduces a residual chemical reagent, which is not conducive to cell growth. The high UV radiation intensity is not conducive to cell loading and growth, and the low radiation intensity cannot form a high-strength hydrogel. Therefore, many problems need to be solved with PVA-based hydrogels. Accordingly, the best solution is to combine physical and chemical methods and explore innovative ways to prepare hydrogels.
